# Direct comparison of PET/CT and MRI to predict the pathological response to neoadjuvant chemotherapy in breast cancer: a meta-analysis

**DOI:** 10.1038/s41598-017-08852-8

**Published:** 2017-08-16

**Authors:** Lihua Chen, Qifang Yang, Jing Bao, Daihong Liu, Xuequan Huang, Jian Wang

**Affiliations:** 1 Department of Radiology, Southwest Hospital, Third Military Medical University, Chongqing, 400038 China; 2 Department of Radiology, PLA No.101 Hospital, Wuxi, Jiangsu Province 214044 China; 3 Department of Radiology, PLA No.44 Hospital, Guiyang, Guizhou Province 550009 China; 4 Molecular biology laboratory, Wuxi center for disease control and prevention, Wuxi, Jiangsu Province 214001 China

## Abstract

Both PET/CT and breast MRI are used to assess pathological complete response to neoadjuvant chemotherapy (NAC) in patients with breast cancer. The aim is to compare the utility of PET/CT and breast MRI by using head-to-head comparative studies. Literature databases were searched prior to July 2016. Eleven studies with a total of 527 patients were included. For PET/CT, the pooled SEN was 0.87 (95% confidence interval (CI): 0.71–0.95) and SPE was 0.85 (95% CI: 0.70–0.93). For MRI, the pooled SEN was 0.79 (95% CI: 0.68–0.87) and SPE was 0.82 (95% CI: 0.72–0.89). In the conventional contrast enhanced (CE)-MRI subgroup, PET/CT outperformed conventional CE-MRI with a higher pooled sensitivity (0.88 (95% CI: 0.71, 0.95) vs. 0.74 (95% CI: 0.60, 0.85), P = 0.018). In the early evaluation subgroup, PET/CT was superior to MRI with a notable higher pooled specificity (0.94 (95% CI: 0.78, 0.98) vs. 0.83 (95% CI: 0.81, 0.87), P = 0.015). The diagnostic performance of MRI is similar to that of PET/CT for the assessment of breast cancer response to NAC. However, PET/CT is more sensitive than conventional CE-MRI and more specific if the second imaging scan is performed before 3 cycles of NAC.

## Introduction

Neoadjuvant chemotherapy (NAC) followed by surgery has been used as a standard treatment and offers advantages over traditional adjuvant approaches in patients with locally advanced breast cancer^1^. It has been established that early response after NAC, achieving pathologic complete response (pCR) or a minimal residual tumour burden might be an optimal predictor of a favorable long-term outcome^2^. Early prediction of outcome and monitoring the response to NAC are important for optimal management by improving the ability to individualise therapies, such as by avoiding additional toxic therapy in non-responding patients^3^.

Various noninvasive imaging tools are used to follow tumour change after NAC, including mammography, ultrasound, and magnetic resonance imaging (MRI). Breast MRI has been increasingly shown to correlate better with pathologic breast tumour size^4^. With the development of quantitative perfusion MRI, diffusion-weighted MRI (DWI) and magnetic resonance spectroscopy (MRS), multiparametric MRI also has been recommended as an accurate biomarker for NAC response evaluation in patients with operable breast cancer^5, 6^. Positron emission tomography (PET) with ^18^F-fluorodeoxyglucose (FDG) is correlated with increased glucose metabolism in cancer. This correlation has been harnessed to evaluate the clinical response to NAC in patients with breast cancer. Metabolic reduction detected between baseline and the early phase of NAC can provide early information on the potential tumour response.

Several systematic reviews have reported the accuracy of breast MRI or ^18^F-FDG PET/CT alone in predicting pathological response to NAC in breast cancer^5, 7, 8^. In addition, a large number of studies^6, 9–23^ have compared the value of MRI and PET/CT directly for the assessment of breast cancer response to NAC against a reference standard of histopathologic analysis. However, the findings of these studies have been inconsistent, and most of their sample sizes were small. Therefore, we conducted a meta-analysis of the literature to estimate the diagnostic performance of breast MRI compared with PET/CT for monitoring response to NAC in breast cancer. To identify the best evidence of the diagnostic performance of these two methods, we restricted the scope of this meta-analysis to direct comparative diagnostic accuracy studies.

## Results

**Figure 1:**
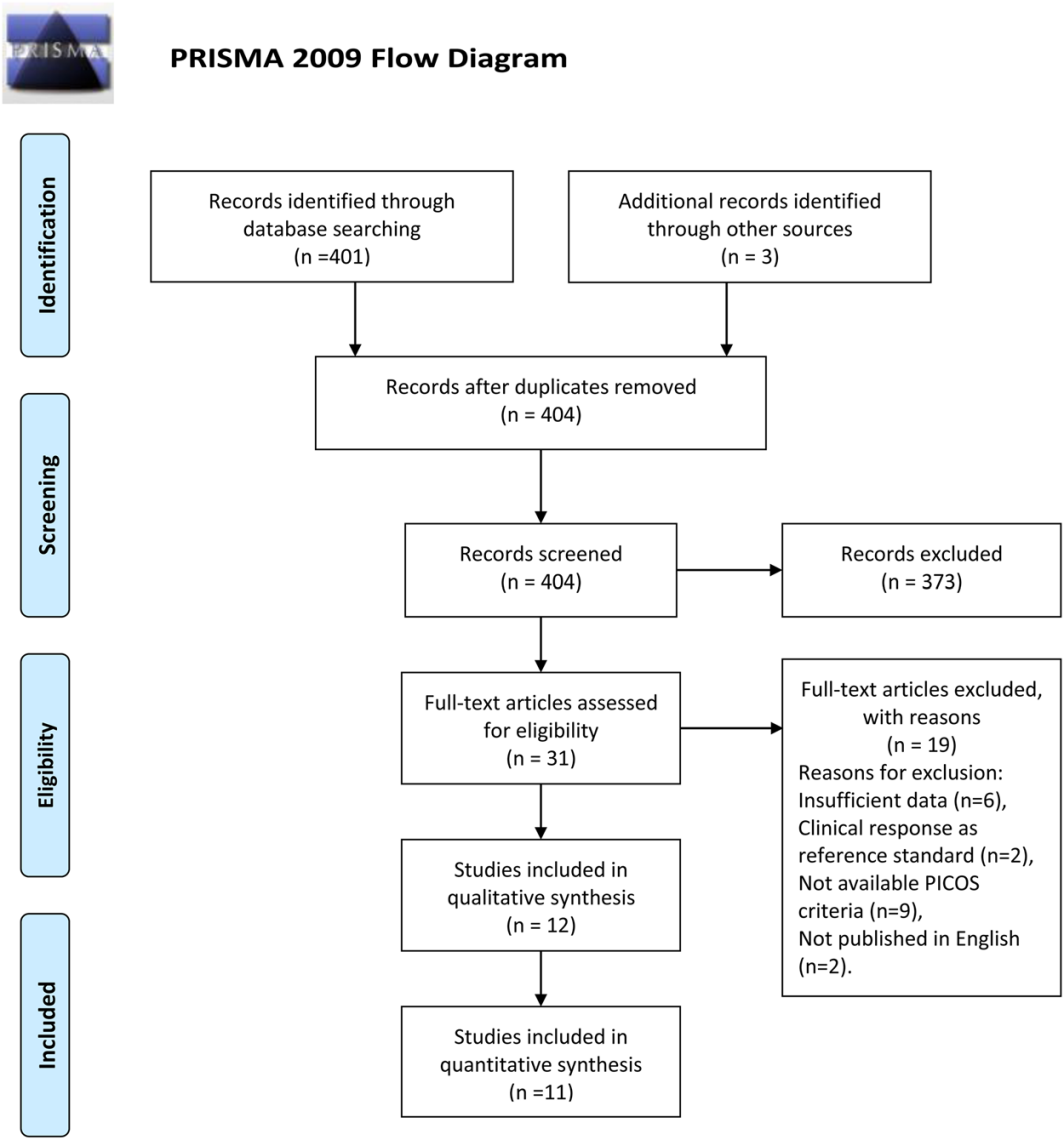
Flowchart illustrating the selection of studies^32^.

The database search initially identified 401 potential literature citations, and 3 additional records were obtained by searching the grey literature (Fig. 1). After reviewing the titles and abstracts, 373 of the studies were excluded as they were not relevant studies. After reading the full texts, we excluded 19 of the remaining 31 articles for the following reasons: 6 article lacked sufficient information to enable completion of a 2 × 2 contingency table, 9 article was not available, the reference standard in 2 articles was clinical response, and 2 article was not published in English. After this final screening, 12 published studies met our inclusion criteria. Ultimately, a total of 11 studies were included in our quantitative synthesis; 1 study was excluded because it assessed the axillary lymph node response to NAC. The data extracted from these individual studies are summarised in Table 1, Table 2, Table S1, and Table S2.

**Table 1:** Summary of the cohort, tumour, and treatment characteristics of the included studies.

Variable		Number providing data		Median estimate	Range
		Studies	Patients		
Cohort characteristics					
No., all tests		12	641	53.4	16–142
Age (years)		12	641	50.5	24–71
pCR (prevalence)		12	245	40.7%	16.9–85.0%
non-pCR (prevalence)		12	396	59.3%	15.0–83.1%
Tumour characteristics					
Stage	I	2	20	11.9%	6.3–17.5%
	II	7	235	42.2%	10.0–68.3%
	III	8	210	58.5%	7.9–90.0%
	IVa	3	9	4.3%	3.4–6.3%
Histology	IDC	8	390	84.5%	39.6–96.4%
	ILC	7	56	14.9%	1.8–58.5%
	MC	5	5	3.1%	1.8–6.2%
	Other	2	3	2.3%	1.1–5.8%
Receptor	ER (+)	5	149	48.0%	4.3–75%
	PR (+)	4	121	51.0%	36.6–68.8%
	HER-2 (+)	6	176	33.6%	17.1–78.2%
	LA	2	10	16.5%	7.9–28.6%
	LB	3	46	54.5%	28.6–100%
	Triple (−)	5	65	24.5%	12.5–33.3%

ER = oestrogen receptor; PR = progesterone receptor; HER2 = human epidermal growth factor receptor 2; IDC = invasive ductal carcinoma; ILC = invasive lobular carcinoma; MC = mucinous carcinoma; LA = luminal A; LB = luminal B; NAC = neoadjuvant chemotherapy; NR = not reported; pCR = pathologic complete response; Triple (−) = Triple negative.

**Table 2:** Absolute numbers of the included studies.

Study	Patient (No.)	Design	Time of scan	MRI				PET/CT			
				Parameter	Cut-off	Sen	Spe	Parameter	Cut-off	Sen	Spe
An, Y 2015	20	Retro	B & A (preoperative)	ΔLD	Reduction > 30%^a^	0.33	0.82	ΔSUV	Reduction > 30%^c^	0.33	0.88
				ΔLD	Increase > 88.7%^b^	0.67	0.94	ΔSUV	Reduction > 80.6%^b^	0.67	0.88
				ΔADC	Increase > 22.1%^b^	0.67	0.71				
Choi, J 2010	41	Pro	B & A (3 or 8 cycles)	ΔLD	Reduction > 30%^a^	0.71	0.95	ΔSUV	Reduction > 50%^d^	0.86	0.38
Kim, T 2014	56	Retro	B & A (3 or 6 cycles)	ΔLD	Reduction > 50%^b^	0.91	0.77	ΔSUV	Reduction > 60%^b^	0.91	0.73
Pahk, K 2015	21	Retro	B & A (3 or 4 cycles)	ΔLD	Reduction > 38%^b^	0.71	0.71	ΔSUV	Reduction > 69%^b^	0.86	1.00
Park, J 2011	32	Retro	B & A (18–22 days)	ΔLD	Reduction > 30%^a^	0.63	0.96	ΔSUV	Reduction > 50%^d^	1.00	0.63
Park, S 2012	34	Retro	B & A (3 or 6 cycles)	ΔADC	Increase > 55%^b^	1.00	0.70	ΔSUV	Reduction > 64%^b^	1.00	0.78
Pengel 2014	93	Pro	B & A (1 or 3 cycles)	ΔLD	Reduction > 50%^b^	0.86	0.58	ΔSUV	Reduction > 50%^b^	0.47	0.94
Tateishi 2012	142	Retro	B & A (2 cycles)	ΔLD	Reduction > 30%^a^	0.46	0.86	ΔSUV	Reduction > 30%^c^	0.67	0.96
				ΔKep	Reduction > 63%^b^	0.52	0.92	ΔSUV	Reduction > 80%^b^	0.70	0.96
Cho, N 2016	35	Pro	B & A (1 cycles)	ΔtCho	Reduction > 61%^b^	1.00	0.76	ΔSUV	Reduction > 62%^b^	0.67	1.00
Amioka 2016	63	NR	B & A (NR)	ΔLD	Reduction > 30%^a^	0.70	0.85	ΔSUV	NR	1.00	0.53
Chen 2004	16	Retro	B & A (NR)	ΔLD	Reduction > 30%^a^	0.90	0.17	ΔSUV	Reduction > 50%^d^	0.90	0.83
				ΔLD	Reduction > 63%^b^	0.90	0.50	ΔSUV	Reduction > 50%^b^	0.90	0.83
Hieken 2013^†^	88	Pro	B & A (NR)	ΔLD	NR	0.61	0.59	ΔSUV	NR	0.63	0.85

^a^Cut-off set by pre-specified RECIST criteria; ^b^cut-off set by ROC analysis; ^c^cut-off set by pre-specified PRECIST criteria; ^d^cut-off set by pre-specified EORTC criteria; B & A, at baseline and after NAC; Pro, prospective; Retro, retrospective; NR, not reported; ΔLD, change in longest diameter; ΔADC, change in apparent diffusion coefficient; ΔKep, change in transfer constant; ΔtCho, change in total choline-containing compounds; ΔSUV, change in standardised uptake values. ^†^Study assessed axillary lymph node response to NAC.

**Figure 2:**
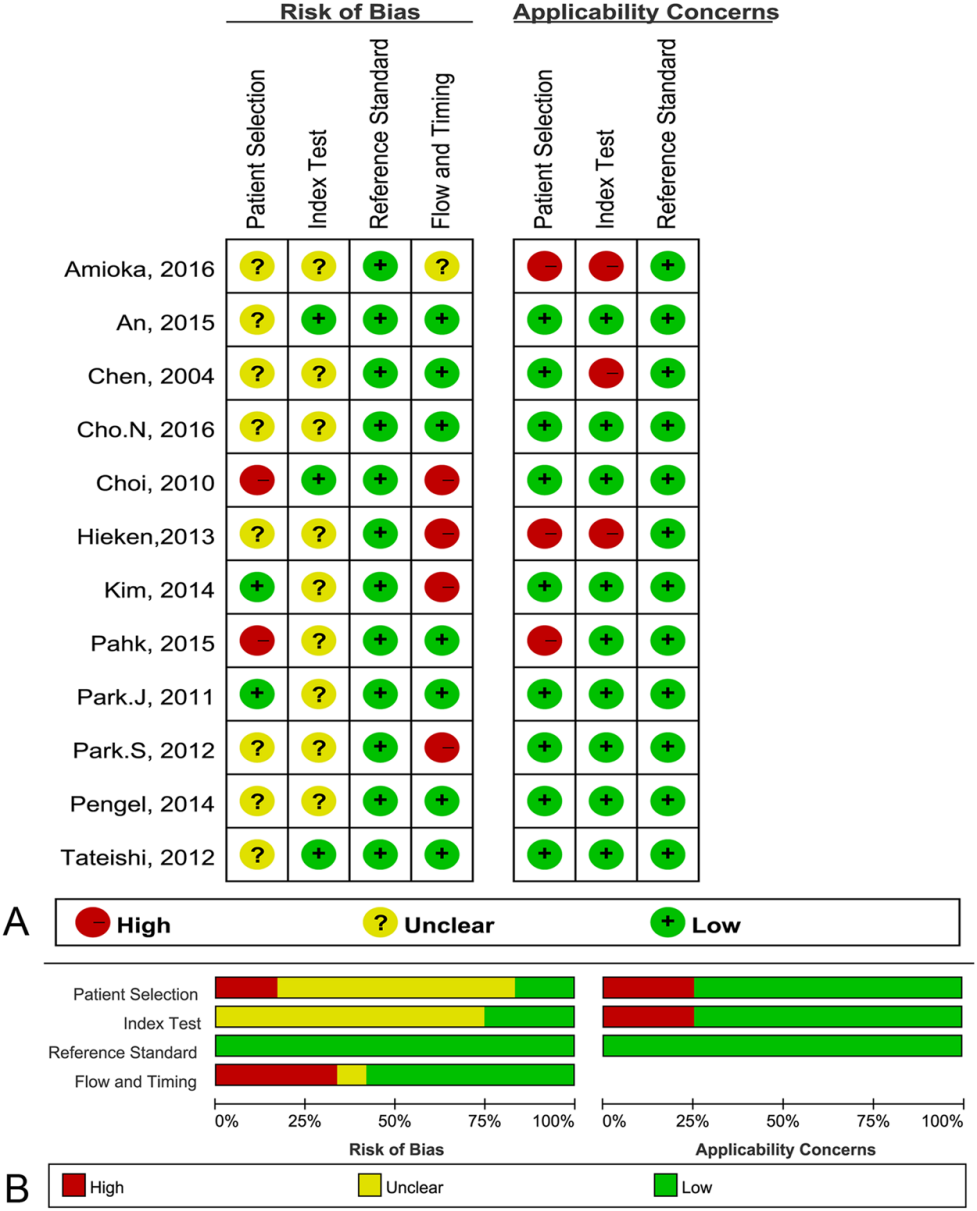
Methodological quality of the 12 included studies. (**A**) Risk of bias and applicability concerns summary; (**B**) risk of bias and applicability concerns graph.

According to QUADAS-2, the quality assessment of the 12 studies was moderate. The results of the distribution of the study design are shown in Fig. 2.

**Figure 3:**
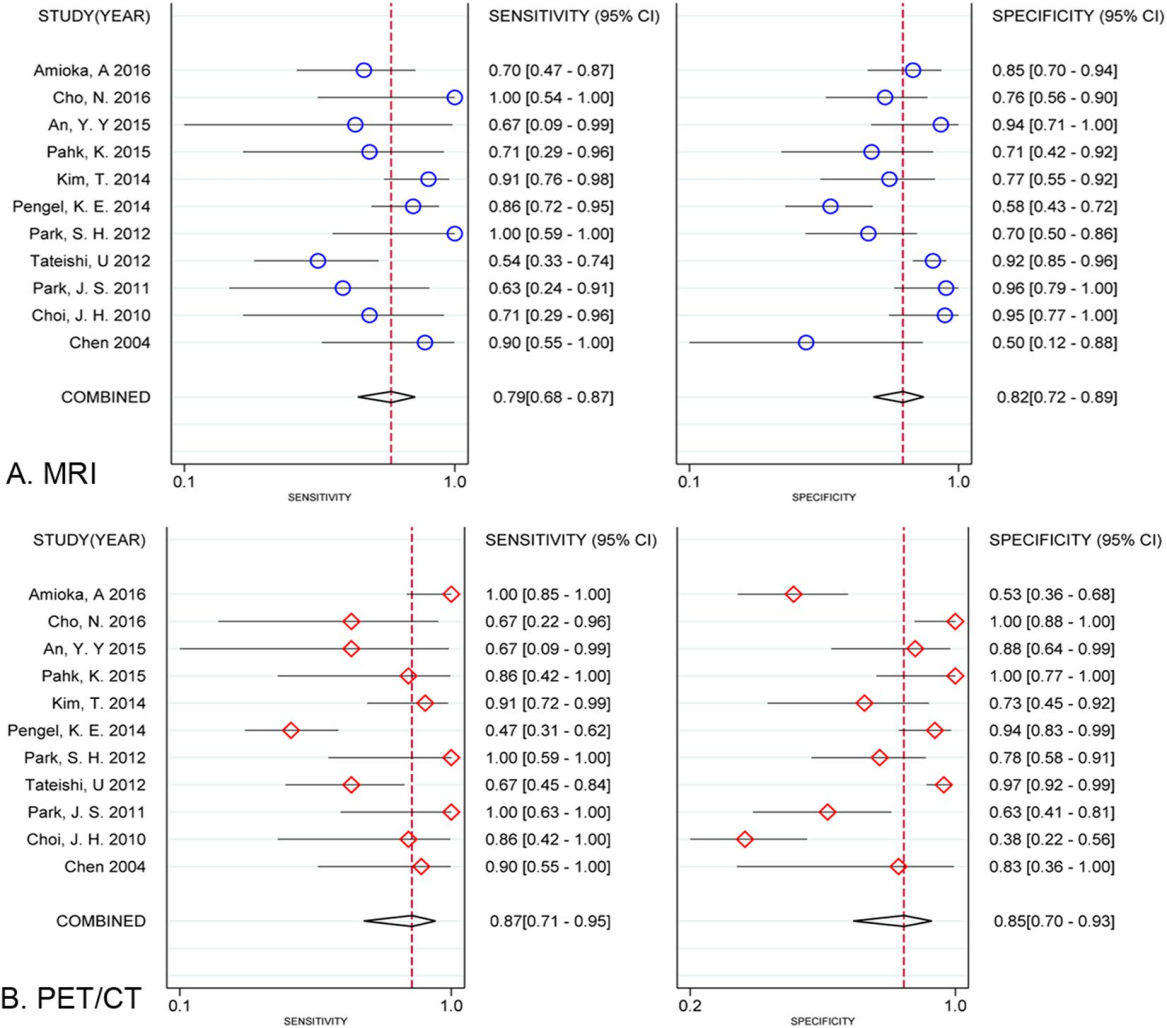
Forest plots of SEN and SPE with corresponding 95% CIs of MRI and PET/CT in assessing pathologic response to NAC. (**A**) MRI; (**B**) PET/CT.

**Figure 4:**
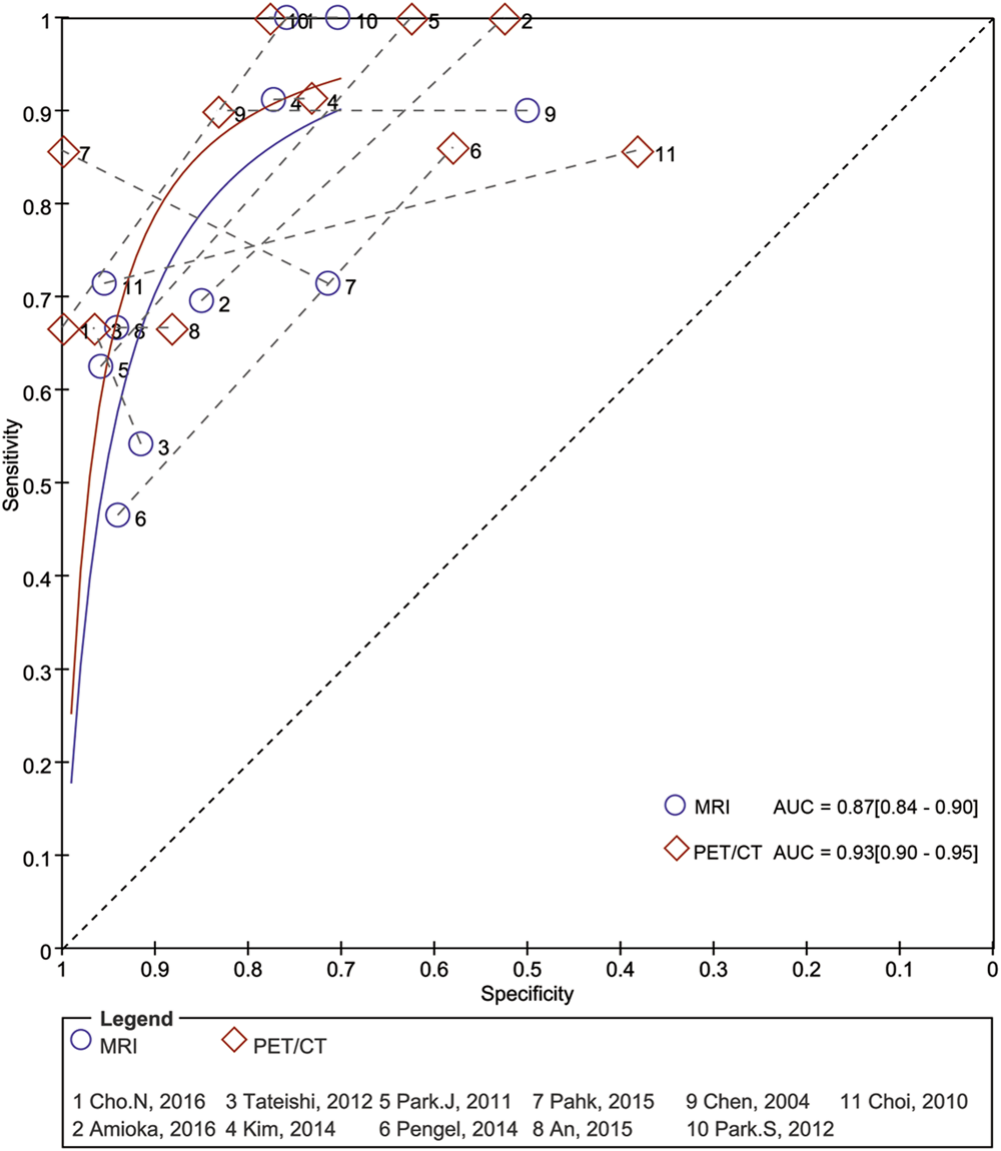
Pairs of observed values of sensitivity and specificity for MRI and PET/CT to assess pathologic response to NAC in HSROC curves.

As there was significant heterogeneity in both pooled analyses (MRI: I^2^ = 92.8%, P < 0.001; PET/CT: I^2^ = 97.2%, P < 0.001), we used a random-effects coefficient binary regression model. The pooled weighted values for MRI were sensitivity (SEN) 0.79 (95% CI: 0.68, 0. 88), sensitivity (SEN) 0.82 (95% CI: 0.72, 0.89), positive likelihood ratio (PLR) 4.29 (95% CI: 2.92, 6.30), negative likelihood ratio (NLR) 0.26 (95% CI: 0.18, 0.39), diagnostic odds ratio (DOR) 16.43 (95% CI: 10.05, 26.87), and the areas under the ROC curve (AUC) 0.87 (95% CI: 0.84, 0.90). The pooled weighted values for PET/CT were SEN 0.87 (95% CI: 0.71, 0. 95), SPE 0.85 (95% CI: 0.70, 0.93), PLR 5.76 (95% CI: 2.96, 11.12), NLR 0.16 (95% CI: 0.07, 0.34), DOR: 37.25 (95% CI: 17.00, 81.62), and AUC 0.93 (95% CI: 0.90, 0.95). The forest plots for the 11 studies are shown in Fig. 3. Hierarchical summary receiver operating characteristic (HSROC) curves are shown in Fig. 4.

The accuracy estimates for the different subgroups are presented in Table 3. In the pre-specified cut-off subgroup, PET/CT outperformed MRI in assessing the pathologic response to NAC, with a higher pooled sensitivity (0.79 [95% CI: 0.65, 0.89] vs. 0.61 [95% CI: 0.39, 0.79], P = 0.005) and a comparable pooled specificity (0.81 [95% CI: 0.75, 0.86] vs. 0.83 [95% CI: 0.54, 0.95], p = 0.713). However, in the cut-off obtained by ROC subgroup, the pooled sensitivity and pooled specificity of PET/CT were similar to those of MRI. In the conventional contrast enhanced (CE)-MRI subgroup, PET/CT was more effective than MRI in assessing the pathologic response to NAC, with a slightly higher pooled sensitivity (0.88 [95% CI: 0.71, 0.95] vs. 0.74 [95% CI: 0.60, 0.85], P = 0.018) and pooled specificity (0.82 [95% CI: 0.65, 0.92] vs. 0.82 [95% CI: 0.71, 0.89], P = 0.999). However, in the functional MRI subgroup, PET/CT appeared to have lower pooled sensitivity (0.78 [95% CI: 0.52, 0.92] vs. 0.88 [95% CI: 0.49, 0.98], P = 0.060) but higher pooled specificity (0.92 [0.82–0.98] vs. 0.82 [0.67–0.89], P = 0.057) than MRI. In the early evaluation subgroup, PET/CT was superior to MRI, with a similar pooled sensitivity (0.71 [95% CI: 0.35, 0.92] vs. 0.73 [95% CI: 0.53, 0.87], P = 0.753) and a notably higher pooled specificity (0.94 [95% CI: 0.78, 0.98] vs. 0.83 [95% CI: 0.64, 0.93], P = 0.015). By contrast, in the post evaluation subgroup, the pooled sensitivity and specificity of PET/CT were very similar to those of MRI.

**Table 3:** Accuracy estimates for subgroup analyses.

Factor	Subgroups	Imaging	No	pSEN (95% CI)	pSPE (95% CI)	AUC (95% CI)
Cut-off value	ROC analysis	MRI	9	0.80 (0.73–0.86)	0.80 (0.75–0.84)	0.86 (0.83–0.89)
		PET/CT	9	0.74 (0.66–0.81)	0.88 (0.84–0.91)	0.92 (0.90–0.94)
				P = 0.313	P = 0.123	P = 0.124
	Pre-specified	MRI	5	0.61 (0.39–0.79)	0.83 (0.54–0.95)	0.73 (0.69–0.76)
		PET/CT	5	0.79 (0.65–0.89)	0.81 (0.75–0.86)	0.87 (0.82–0.92)
				P = 0.005*	P = 0.713	P = 0.022*
MRI modality	Conventional CE-MRI	MRI	9	0.74 (0.60–0.85)	0.82 (0.71–0.89)	0.84 (0.81–0.87)
		PET/CT	9	0.88 (0.71–0.95)	0.82 (0.65–0.92)	0.92 (0.89–0.94)
				P = 0.018*	P = 0.999	P = 0.104
	Functional MRI	MRI	4	0.87 (0.49–0.98)	0.82 (0.67–0.89)	0.89 (0.86–0.91)
		PET/CT	4	0.78 (0.52–0.92)	0.92 (0.82–0.98)	0.93 (0.90–0.95)
				P = 0.060	P = 0.057	P = 0.258
Evaluation time	Early evaluation	MRI	4	0.73 (0.53–0.87)	0.83 (0.64–0.93)	0.85 (0.81–0.88)
		PET/CT	4	0.71 (0.35–0.92)	0.94 (0.78–0.98)	0.92 (0.89–0.94)
				P = 0.753	P = 0.015*	P = 0.163
	Post evaluation	MRI	5	0.85 (0.68–0.94)	0.83 (0.70–0.92)	0.91 (0.88–0.93)
		PET/CT	5	0.89 (0.77–0.96)	0.80 (0.53–0.93)	0.90 (0.87–0.93)
				P = 0.400	P = 0.585	P = 0.798

pSEN = pooled sensitivities; pSPE = pooled specificities; *P < 0.05.

**Figure 5:**
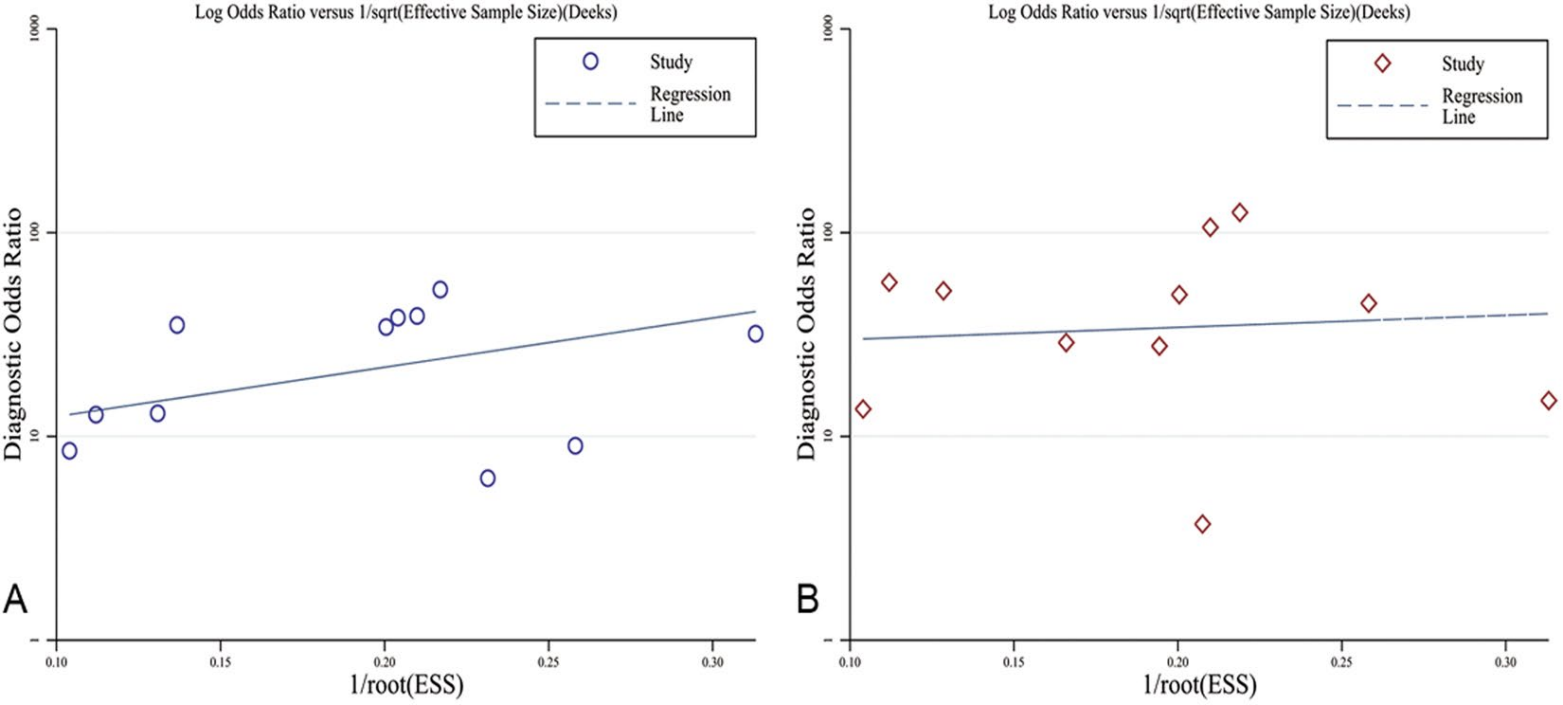
Funnel plot of publication bias. (**A**), MRI P = 0.160; (**B**) PET/CT P = 0.804.

The results of Deeks funnel plot asymmetry test (P = 0.160 and P = 0.804, respectively) showed no evidence of notable publication bias in the analysis of either MRI or PET/CT (Fig. 5).

## Discussion

Although MRI and PET/CT already play daily clinical roles in determining whether to continue, change, or abandon NAC for breast cancer, previous meta-analyses and systematic reviews have yielded inconsistent findings^7, 8, 24^ when assessing these imaging modalities alone or together (Table 4). Several recent head-to-head comparative studies have also yielded inconsistent findings^13, 17, 21, 22^. Because head-to-head comparisons provide the best measurements of the diagnostic accuracy of two different techniques^25, 26^, we focused exclusively on direct comparative studies that evaluated both MRI and PET/CT in the same cohort of patients. Compared with the previous meta-analysis by Liu^24^, our research is strengthened by more careful selection of articles and the inclusion of two direct comparative studies^6, 15^ that might be missed in their analysis.

**Table 4:** Summary of meta-analyses focused on MRI and PET/CT for the assessment of breast cancer response to NAC.

Study	Search date	No.	Modality	PSEN (95% CI)	PSPE (95% CI)	DOR (95% CI)	AUC (95% CI)
Michae l^7^	to 2011	44	MRI	0.92(0.85–0.97)	0.60(0.39–0.96)	17.89(11.45, 27.95)	0.88(NR)
Mghanga^8^	2000–2012	15	PET/CT	0.81(0.76, 0.85)	0.79(0.74, 0.83)	NR	0.88(0.86–0.90)
Liu^24^	1992–2015	6	MRI	0.65(0.45, 0.80)	0.88(0.75, 0.95)	NR	0.84(0.80, 0.87)
		6	PET/CT	0.86(0.76, 0.93)	0.72(0.49, 0.87)	NR	0.88(0.85, 0.91)
Our	2000–2016	11	MRI	0.79(0.76, 0.87)	0.82(0.72, 0.89)	16.43(10.05, 26.87)	0.87(0.84, 0.90)
		11	PET/CT	0.87(0.71, 0.95)	0.85(0.70, 0.93)	37.25(17.01, 81.62)	0.93(0.90, 0.95)

PSEN = pooled sensitivities; PSPE = pooled specificities; DOR = diagnostic odds ratio; NR = not reported.

The results of our meta-analysis showed that MRI and PET/CT have similar high sensitivities (0.79 vs. 0.87) and specificities (0.82 vs. 0.85). However, among previous meta-analyses, the study focusing on MRI by Michael *et al*.^8^ had a much higher pooled sensitivity (0.92 vs. 0.81) than the study focusing on PET/CT by Mghanga *et al*.^7^, whereas completely opposite results were observed for pooled specificity (0.60 vs. 0.79). In addition, the AUCs of the two studies were identical (0.88 vs. 0.88). We speculate that the characteristic of high sensitivity with low specificity or vice versa may be caused by a threshold effect originating from the use of different diagnostic cut-off values in various studies. Due to this threshold effect, ROC curve and AUC analysis are more insightful approaches than evaluating the pooled sensitivity and pooled specificity. The AUC in our study (0.87 vs. 0.93) is consistent with these meta-analyses, which suggests that the diagnostic performance of MRI is similar to that of PET/CT for the assessment of breast cancer response to NAC.

Traditionally, tumour response has been monitored by conventional CE-MRI alone with standard anatomic response criteria (Response Evaluation Criteria in Solid Tumors (RECIST) and RECIST 1.1) during the course of NAC imaging^4^. Over the last decade, advances in functional imaging have enabled anatomic and functional information to be obtained, such as by PET/CT, DWI, perfusion MRI, and MRS. On the basis of PET, metabolic response criteria were established, including the European Organisation for Research and Treatment of Cancer (EORTC) criteria and PET Response Criteria in Solid Tumors (PERCIST)^27^. Several studies^13, 14, 20, 23^ attempted to compare the predictive roles of MRI and PET/CT during NAC using a pre-specified cut-off according to international standards (RECIST vs. PERCIST). Therefore, we performed subgroup analysis of different diagnostic cut-off values. In the pre-specified cut-off subgroup, PET/CT outperformed MRI in assessing pathologic response to NAC, with a higher pooled sensitivity (0.79 vs. 0.61) and a comparable pooled specificity (0.81 vs. 0.83). However, this trend was not observed in the cut-off obtained by ROC subgroup. We also performed subgroup analysis of different MRI modalities. In the conventional CE-MRI subgroup, PET/CT was more effective than MRI in assessing pathologic response to NAC, with a slightly higher pooled sensitivity (0.88 vs. 0.74) and pooled specificity (0.82 vs. 0.82). However, in the functional MRI (perfusion MR, DWI, or MRS) subgroup, PET/CT appeared to be equivalent to MRI, with lower pooled sensitivity (0.78 vs. 0.88), higher pooled specificity (0.92 vs. 0.82), and similar AUC (0.93 vs. 0.89). These results suggest that PET/CT is more accurate than conventional CE-MRI imaging and that PERCIST criteria may be more appropriate than RECIST criteria for monitoring breast cancer response to NAC. A possible explanation is the general limitation of anatomic MRI techniques, which are unable to distinguish potential residual tumour from fibrotic scar tissue in stable disease^14^.

Because the delay time between the initiation of therapy and changes in tumour size is usually longer than 2 cycles of NAC^28^, several studies^18–20, 22^ have attempted to investigate earlier predictors associated with angiogenesis, metabolism, or cellularity that may change before tumour shrinkage in the breast cancer response to NAC. Moreover, there is no consensus on the optimal timing of second imaging for evaluation of the response to NAC. Therefore, we performed a subgroup analysis of different evaluation time points of second imaging. In the early evaluation subgroup, PET/CT was superior to MRI in assessing pathologic response to NAC, with a notably higher pooled specificity (0.94 vs. 0.83) and a similar pooled sensitivity (0.71 vs. 0.73). By contrast, in the post evaluation subgroup, the pooled sensitivity, specificity and AUC of PET/CT were very similar to those of MRI. Our results support previous conjecture that PET/CT is superior to MRI in assessing response at times before 3 cycles of NAC but not at times after 3 cycles of NAC.

Although breast surgical resection after NAC is based on a combination of clinical and imaging assessments of the response to treatment, the axillary nodal stage continues to play a crucial role in clinical decisions. Hieken *et al*.^15^ reported that PET/CT has higher sensitivity (0.86 vs. 0.59) than MRI in assessing the axillary lymph node response to NAC. However, this result must be interpreted with caution because only one study of this type is available. More clinical studies are required to confirm this result, which would indicate that PET/CT has a greater advantage in assessing both breast cancer and axillary lymph node response to NAC than MRI.

The performance of either PET/CT or MRI alone was shown much different among breast cancer subtypes. Therefore, imaging techniques based on subtypes for personalizing may further improve their performance in NAC monitoring^29^. However, after reviewing the 12 included articles, only two studies with knowledge of the breast cancer subtypes were identified in our study (Table S2). One head-to-head comparative study revealed that it might be better to use PET/CT for early predicting pCR than conventional CE-MRI in luminal B subtype breast cancer^17^. The second study showed that pCR was associated with the reduction in SUVmax on PET/CT as well as the reduction in largest diameter on MRI in triple-negative tumours, but not in HER2-positive and ER-positive/HER2-negative tumours^19^. Although current evidence is not sufficient to draw recommendations, these results may be clinically useful and generate hypotheses for further research.

Some intrinsic disadvantages of our study should be considered when interpreting our results. First, the sample sizes of comparative studies available in the literature are relatively small, which may contribute to an overestimation of diagnostic accuracy^26^. However, a systematic review^30^ focused on meta-analysis studies from the Cochrane Database showed that the number of studies eligible for meta-analysis is typically small in all medical areas and for all outcomes and interventions covered by the Cochrane Reviews. Second, there may be publication bias in this meta-analysis. Our meta-analysis was based only on published and full-text articles, which tend to report positive or significant results rather than negative or not significant results. Although the quality of published data in peer-reviewed journals is generally considered superior to unpublished data^31^, the inclusion of only published studies may lead to reporting bias. Third, accuracy estimates are affected by various factors, such as the definition of pCR and the breast cancer phenotype^8^. As data are limited to investigate those factors, we did not assess these factors in our analyses.

In conclusion, a limited number of head-to-head studies indicates that the diagnostic performance of MRI is similar to that of PET/CT for the assessment of breast cancer response to NAC. However, for monitoring breast cancer response to NAC, PET/CT is more sensitive than anatomic MR imaging, and PERCIST criteria may be more appropriate than RECIST criteria. Moreover, PET/CT is superior to MRI in assessing response at times between 1–3 cycles of NAC but not at time after 3 cycles of NAC. In the future, large-scale, head-to-head, well-designed trials are necessary to compare the predictive value and consider more factors (such as the definition of pCR and phenotype of breast cancer) of these two imaging techniques.

## Materials and Methods

We used the Preferred Reporting Items for Systematic Reviews and Meta-Analyses statement^32^ to improve the reporting of our research (Fig. 1).

### Search Strategy

A structured approach was followed to identify the patient population, interventions, comparators, outcomes, and study design (PICOS criteria)^32^. Two observers (Lihua Chen and Qifang Yang) performed the literature search of data sources independently (PUBMED, EMBASE, Web of Science, and the Cochrane Library). The search strategy (Appendix A) included both subject headings (MeSH terms) and keywords for the target condition (breast cancer), the imaging techniques under investigation (MRI and PET/CT), and the interventions (neoadjuvant therapy). We limited our search to publications with the search term in the title or abstract of the article and a publication date no later than July 2016. Review articles, letters, comments, case reports, and unpublished articles were excluded. Extensive cross-checking of the reference lists of all retrieved articles was performed.

### Criteria for inclusion in the study

Studies were eligible if the following PICOS criteria were met. (a) The patient population consisted of primary breast cancer confirmed histologically; (b) the imaging response for pre-NAC and post-NAC was monitored with both MRI and FDG-PET; (c) histopathologic analysis was available as a reference standard; (d) the study outcome described pCR or near-pCR to NAC; and (e) the study design was described as a direct comparative study or randomised controlled trial.

Non-English and non-Chinese articles were excluded if a full-text translation or evaluation could not be obtained. Both prospective and retrospective studies were included.

We excluded studies if a 2 × 2 table could not be extracted from the data, if there were fewer than 10 patients, and if multiple reports were published for the same study population. In the latter case, the most detailed or recent publication was extracted.

### Selection of Articles

Articles were selected by two authors (Lihua Chen and Qifang Yang) independently. The two authors initially screened the titles and abstracts of the search results and retrieved all potentially relevant reports in full. Next, they reviewed all relevant reports according to the predefined inclusion criteria. Disagreements were arbitrated by a third author (Jian Wang) who assessed all involved items.

### Quality Assessment and Data Extraction

For each included study, the methodological quality was evaluated independently by two observers (Lihua Chen and Qifang Yang) using the standard quality assessment of diagnostic studies (QUADAS-2) checklist, which was specifically developed for systematic reviews of diagnostic accuracy studies^33–35^. In addition, the relevant information was also extracted from each study, including the author, year of publication, description of the study population, study nation, study design characteristics, therapeutic interventions, reference standard, evaluation time, and descriptions of the interpretations of the diagnostic tests. The true-positive (TP), false-positive (FP), true-negative (TN), and false-negative (FN) data were extracted or derived to construct 2 × 2 contingency tables.

### Meta-analysis

We constructed forest plots to show the variations of the SEN and SPE estimates together with 95% confidence intervals (CI) for each imaging test in each study. We calculated the SEN, SPE, PLR, NLR and DOR values with their 95% CIs. We constructed HSROC curves to estimate SEN and SPE^36^.

Standard χ2-testing and the inconsistency index (I-squared, I^2^) were used to assess the heterogeneity of the individual studies using Stata software (Stata Corporation, College Station, TX, USA). P < 0.1 or I^2^ > 50% suggested notable heterogeneity^37^. If notable heterogeneities were detected, the test performance was summarised using a random-effects coefficient binary regression model; otherwise, a fixed-effects coefficient binary regression model was used^25^.

Subgroup analyses were performed as follows: (a) comparisons of studies using different cut-off values: ROC analysis subgroup (cut-off obtained by ROC analysis) or pre-specified subgroup (cut-off set by pre-specified criteria, MRI with anatomic response criteria, and PET/CT with metabolic response criteria); (b) comparisons of studies using different MRI modalities: conventional CE-MRI subgroup (longest diameter or tumour volume) or functional MRI subgroup (parameter of quantitative perfusion MR, DWI, or MRS); and (c) comparisons of studies with different evaluation time points of second imaging: early evaluation subgroup (second imaging scan before 3 cycles) or post evaluation subgroup (second imaging scan after 3 cycles).

The presence of publication bias was assessed by a Deeks funnel plot and an asymmetry test. Publication bias was considered present if there was a nonzero slope coefficient (P < 0.05), which suggests that only small studies reporting high accuracy had been published^38, 39^.

## Electronic supplementary material

Supplementary information
