# Supplementary material for: Direct comparison of PET/CT and MRI to predict the pathological response to neoadjuvant chemotherapy in breast cancer: a meta-analysis
Source: Sci Rep. 2017 Aug 16;7:8479. doi: 10.1038/s41598-017-08852-8 (PMC5559519; doi:10.1038/s41598-017-08852-8)
Supplement: Supplementary file 1 — Supplementary information [file 41598_2017_8852_MOESM1_ESM.doc]

**Title Page**

**Title:** Direct comparison of PET/CT and MRI to predict the pathological response to neoadjuvant chemotherapy in breast cancer: a meta-analysis.

**Authors:** Lihua Chen^1,2^, †MS; Qifang Yang^1,3^, †MS; Jing Bao^4^, MS; Daihong Liu^1^, MS; Xuequan Huang^1^, *MD; Jian Wang^1^, *MD

^1^Department of Radiology, Southwest Hospital, Third Military Medical University, Chongqing, 400038, China

^2^Department of Radiology, PLA No.101 Hospital, Wuxi, Jiangsu Province, 214044, China

^3^Department of Radiology, PLA No.44 Hospital, Guiyang, Guizhou Province, 550009, China

^4^Molecular biology laboratory, Wuxi center for disease control and prevention, Wuxi, Jiangsu Province, 214001, China

† Lihua Chen and Qifang Yang contributed equally to this work

* Co-corresponding Author

**Correspondence to**:

Jian Wang

Department of Radiology,

Southwest Hospital,

Third Military Medical University,

Chongqing, 400038, China

Tel: +86-23-6875-4419

Fax: +86-23-6546-3026

E-mail: [wangjian811@gmail.com](mailto:wangjian811@gmail.com)

**Or**

Xuequan Huang

Department of Radiology,

Southwest Hospital,

Third Military Medical University,

Chongqing, 400038, China

Tel: +86-23-6875-4419

Fax: +86-23-6546-3026

E-mail: [hxuequan@163.com](mailto:hxuequan@163.com)

**Supporting Information Legends**

**Table S1.** Summary of cohort, tumor stage, histologic type, treatment regimens, pathologic response classification and imaging characteristics of included studies

**Table S2.** Absolute numbers of included studies on breast cancer subtypes

**Appendix A.** Search string

**Table S1. Summary of cohort, tumor stage, histologic type, treatment regimens, pathologic response classification and imaging characteristics of included studies***

| **Study** | **Year** | **Stage(No.)** | **Histologic type**  **(No.)** | **Receptor(No.)** | **NAC regimens (No.)** | **Pathologic response classification (No.)** | **MR and Field** | **Modality of MR (Parameter)** | **PET/CT** |
| --- | --- | --- | --- | --- | --- | --- | --- | --- | --- |
| **An, Y. Y** | 2015 | II (2), III (18) | IDC (19), ILC (1) | LA (5), LB (7),TN (3), HER21 (5) | [Dox+Doc](13), [Dox+Cyc](6), [(Dox+Doc)/(Dox+Cyc)] (1) | RCB Index: (0,1)=pCR, (2,3)=non-pCR | Siemens 1.5T | CE-MRI(ΔLD), DWI(ΔADC)**†** | Siemens PET/CT |
| **Choi, J. H.** | 2010 | Ⅱ(14), Ⅲ(27) | IDC (36), ILC (2), MC (1), Other (2) | ER(+) (19), PR(+) (15), HER-2(+) (10) | [Adr+Cyc 4](31), [Pac+Gem+Tra 6](3) [(Adr+Cyc)4+Doc 4](3), [Adr+Doc 3](4), | pCR and non-pCR (Definition not specified) | NR | CE-MRI(ΔLD) | NR, PET/CT |
| **Hieken, T. J.** | 2013 | Ⅰ, Ⅱ, Ⅲ | IDC、ILC、MC、other | NR | NR | NR | GE 1.5T | CE-MRI(ΔLD) | GE PET/CT |
| **Kim, T.** | 2014 | II (24), III (33), IVa (2) | IDC (54), ILC (1), MC (1) | NR | [Tax+Ant 3or 6] (48), Ant 3or 6 (11) | Miller-Payne Grading System: (3-5)= pCR, (1-2)=non-pCR | GE 1.5T or Philips 3.0T | CE-MRI(ΔLD) | GE PET/CT |
| **Pahk, K.** | 2015 | III (21) | IDC (21) | LB (21) | [Epi+Doc 6](18), [(Doc+Cyc)4+Doc 4] (3) | pCR and non-pCR (pCR: No invasive cancer, no DCIS) | Siemens 3.0T | CE-MRI(ΔLD) | Philips PET/CT |
| **Park, J. S.** | 2011 | NR | IDC (31), MC (1) | ER(+) (14), PR(+) (13) | [Dox+Doc 3 or 6] (27),[Pac+Gem+Tra 6](5) | pCR and non-pCR (pCR: No invasive cancer, but DCIS may be present) | Philips 1.5T | CE-MRI(ΔLD) | Philips PET/CT |
| **Park, S. H.** | 2012 | NR | IDC (32), MC (1), Mixed (1) | NR | [Dox+Doc 3] (23), [Pac+Gem+Tra 6] (11) | pCR and non-pCR (pCR: No invasive cancer, but DCIS may be present) | GE 1.5T | DWI(ΔADC)**†** | Philips PET/CT |
| **Pengel, K. E.** | 2014 | II (49), III (41), IVa (3) | IDC(85), ILC(7), other(1) | HER-2(+)(25), ER(+)/PR(−)(4), Triple(−)(28) | [Pac+Tra+Car 3](25), [(Dox+Cyc)3 +(Doc+Cap) 3] (68) | pCR and non-pCR (pCR: near pCR, MRD) | Philips 3.0T | CE-MRI(ΔLD) | Philips PET/CT |
| **Tateishi, U** | 2012 | Ia (9), II (95), III (38) | IDC (131), ILC (11) | ER(+) (100) PR(+) (82) HER-2(+) (111) | [Dox+Cyc+Pac] (28) , [FEC+Pac+Tra] (5), [FEC+Pac] (105), [FEC+Doc] (4) | pCR and non-pCR (pCR: near pCR, MRD) | Siemens 3.0T | DCE-MRI(ΔLD and ΔKep) | Siemens PET/CT |
| **Cho, N.** | 2016 | Ⅱ(8)、Ⅲ(27) | NR | HR(+) (18), HER-2(+) (6), Triple(-) (11) | [Dox+Doc 6] (NR), [Dox+Cyc]4+ Doc 4] (NR) | Miller-Payne Grading System: (3-5)= pCR, (1-2)=non-pCR | Siemens 3.0T | MRS(ΔtCho) | Siemens PET/CT |
| **Amioka, A** | 2016 | T1(11),T2(43), T3(5), T4(4) | NR | LA (5), LB (18), HER-2(+) (19), Triple(-) (21) | [(Tax+Ant) 4+ FEC 4] (44),[(Tax+Ant) 4+ FEC 4 + Tra] (19) | pCR and non-pCR (pCR: No invasive cancer, but DCIS may be present) | NR | CE-MRI(ΔLD) | NR, PET/CT |
| **Chen** | 2004 | LABC | IDC (12), ILC (3), MC (1) | ER(+) (12), PR(+) (11), HER-2(+) (2) | [Adr+Cy] (11),[Tax+Adr+Cy] (2), Adr (1),FEC (1) | pCR and non-pCR (pCR: No invasive cancer, but DCIS may be present) | GE 1.5T | CE-MRI(ΔLD) | GE  PET |

* Abbreviations: Adeno, adenocarcinoma; Adr, adriamycin; Ant, anthracycline; Cap, capecitabine; Car, carboplatin; Cy, Cytoxan; Cyc, cyclophosphamide; DCIS, ductal carcinoma in situ; Doc, docetaxel; Dox, doxorubicin; Epi, epirubicin; ER, estrogen receptor; FEC, 5-flourouracil+epirubicin+cyclophosphamide; Gem, gemcitabine; HER2, humanepidermal growth factor receptor 2; IDC, invasive ductal carcinoma; ILC, invasive lobular carcinoma; IMC, invasive micropapilary carcinoma; LA, luminal A; LABC, locally advanced breast cancer; LB, luminal B; MRD, minimal residual disease; MC, mucinous carcinoma; NAC, neoadjuvant chemotherapy; NR, not reported; Pac, paclitaxel; pCR, pathologic complete response; PR, progesterone receptor; RCB, residual cancer burden; Tax, taxane; Tra, trastuzumab; ΔLD, change in longest diameter; ΔADC, change in apparent diffusion coefficient; ΔKep, change in transfer constant; ΔtCho, change in total choline-containing compounds.

**†** b values of 0 and 750 s/mm^2^.

**Appendix A . Search String**

***MeSH Search String***

(((Magnetic Resonance Imaging [MeSH Terms]) AND Positron-Emission Tomography [MeSH Terms]) AND Breast Neoplasms [MeSH Terms]) AND Neoadjuvant Therapy [MeSH Terms])

***Free-Text Search String***

((((((((((((((((((((((((MR imaging) OR magnetic resonance imaging) OR MR) OR magnetic resonance) OR NMR) OR nuclear magnetic resonance imaging) OR MRT) OR magnetic resonance tomography) OR diffusion MR imaging) OR diffusion) OR DWI) OR diffusion weighted imaging) OR diffusion-weighted imaging)) AND (((PET) OR positron emission tomography) OR PET Scan) OR PET Scans) AND ((((((((((breast neoplasm) OR breast tumor) OR Breast Cancer) OR breast carcinoma) OR mammary neoplasm) OR mammary carcinomas) OR mammary cancer) OR cancer of breast) OR malignant neoplasm of breast) OR malignant tumor of breast) cancer of the breast) AND (((neoadjuvant therapy) OR neoadjuvant chemotherapy) OR neoadjuvant treatment)
